# Mechanism and Pharmacodynamic Substance Basis of Raw and Wine-Processed *Evodia rutaecarpa* on Smooth Muscle Cells of Dysmenorrhea Mice

**DOI:** 10.1155/2023/7711988

**Published:** 2023-06-01

**Authors:** Yeqian Liu, Hong Li, Lei Chen, Hongxia Zhao, Jian Liu, Shan Gong, Danfeng Ma, Chunming Chen, Shuiqing Zeng, Hongping Long, Weiqiong Ren

**Affiliations:** ^1^Department of Pharmacy, The First Hospital of Hunan University of Chinese Medicine, No. 95 Shaoshan Middle Road, Changsha, Hunan Province, China; ^2^Department of Pharmacy, The Second People's Hospital of Anhui Province, No. 1868 Dangshan Road, Hefei, Anhui Province, China; ^3^Center for Medical Research and Innovation, The First Hospital of Hunan University of Chinese Medicine, No. 95 Shaoshan Middle Road, Changsha, Hunan Province, China; ^4^Department of Pharmacy, The Children's Hospital of Hunan Province, No. 86 Ziyuan Road, Changsha, Hunan Province, China

## Abstract

**Objectives:**

*Evodia rutaecarpa* (ER) is a well-known herbal Chinese medicine traditionally used for analgesia in dysmenorrhea, headaches, abdominal pain, etc. Notably, the analgesic effect of wine-processed* Evodia rutaecarpa* (PER) was more potent than that of raw ER. This research aimed to investigate the mechanism and pharmacodynamic substance basis of raw ER and PER on smooth muscle cells of dysmenorrhea mice.

**Methods:**

Metabolomics methods based on UPLC-Q-TOF-MS were utilized to analyse the differential components of ER before and after wine processing. Afterwards, the uterine smooth muscle cells were isolated from the uterine tissue of dysmenorrhea and normal mice. The isolated dysmenorrhea uterine smooth muscle cells were randomly divided into four groups: model group, 7-hydroxycoumarin group (1 mmol/L), chlorogenic acid (1 mmol/L), and limonin (50 *μ*mol/L). The normal group consisted of the isolated normal mouse uterine smooth muscle cells, which were repeated 3 times in each group. The cell contraction and the expression of P2X3 and Ca^2+^ in vitro were determined using immunofluorescence staining and laser confocal; ELISA was used for detection of PGE2, ET-1, and NO content after 7-hydroxycoumarin, chlorogenic acid, and limonin administered for 24 h.

**Results:**

The metabolomics results suggested that seven differential compounds were identified in the extracts of raw ER and PER, including chlorogenic acid, 7-hydroxycoumarin, hydroxy evodiamine, laudanosine, evollionines A, limonin, and 1-methyl-2-[(z)-4-nonenyl]-4 (1H)-quinolone. The in vitro results showed that 7-hydroxycoumarin, chlorogenic acid, and limonin were able to inhibit cell contraction and PGE2, ET-1, P2X3, and Ca^2+^ in dysmenorrhea mouse uterine smooth muscle cells and increase the content of NO.

**Conclusion:**

Our finding suggested that the compounds of the PER were different from those of the raw ER, and 7-hydroxycoumarin, chlorogenic acid, and limonin could improve dysmenorrhea in mice whose uterine smooth muscle cell contraction was closed with endocrine factors and P2X3-Ca^2+^ pathway.

## 1. Introduction

Herbal processing, a unique pharmaceutical technology, was originated from the theory of traditional Chinese medicine (TCM) [1]. After processing, there are many noticeable changes in TCM, manifested in the significant difference in the characteristics, channel tropism, chemical components, and treatment effect of all types of TCM, which can cause changes in side effect reduction and therapeutic efficacy [2, 3]. Wine, as an auxiliary material, has been adopted in the processing of TCM for several centuries and strengthens the effect of “warming yang” and dispels cold to relieve pain [4, 5].

*Euodia rutaecarpa (Juss.) Benth. Var. Bodinieri (node) Huang* (ER) is commonly used to improve the analgesic effects in the Pharmacopoeia of the People's Republic of China [6, 7]. It has been proven to have anti-inflammatory and analgesic effects [8, 9]. The PER is the stir-fried product of ER macerated with rice wine, which is recorded in the Essential Recipes for Emergent Use Worth a Thousand Gold [10]. Research has shown that the pharmacological actions of raw ER are different from those of its processed products [11]. Raw ER may dispel cold to relieve pain, and the effect of dispelling cold and relieving pain is strengthened after wine processing [11, 12]. Our prior study demonstrated that raw ER could reduce the number of body twists, prolong the latency of body twisting, and reduce pain in dysmenorrhea mice, and that the effect of PER on dysmenorrhea is better than that of raw ER [11]. Chinese herbal remedies often contain multiple components [13]. However, whether wine processing causes changes in components of raw ER, whether the analgesic action of these components improves dysmenorrhea, and the mechanisms are unknown. Therefore, the components of raw ER and PER and which of them have the potential to exert treatment effects are also a focus of research.

TCM usually contains various chemical compositions; thus, data on their treatment effects, mechanisms of action, and toxicology characteristics are frequently limited [14]. Therefore, it becomes the focus of the research concerning the identification of chemical compositions of such medicines, evaluation of biological, and standardization of safety quality [15]. Plant metabolomics is a comprehensive method that can qualitatively and quantitatively analyse metabolite data in a specific period or condition, and it is combined with chemical informatics analysis to ensure target differential metabolites [16]. UPLC-Q-TOF-MS is a widely used metabolite analysis method because of its high resolution and sensitivity [17]. ER is closely related to dysmenorrhea. A dysmenorrhea mouse uterine smooth muscle cell model was performed to prove the differences between raw ER and PER in terms of their curative effect [18].

**Figure 1:**
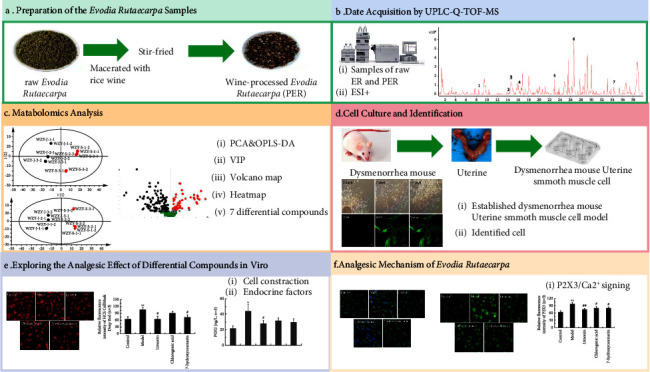
Research conducted in this study. This study was divided into six main parts. (a) Preparation of *Evodia rutaecarpa* samples. (b) Date acquisition of HPLC-Q-TOF-MS. (c) Metabolomics analysis. (d) Cell culture and identification. (e) Exploring the analgesic effects of differential compounds in vitro. (f) Analgesic mechanism of *Evodia rutaecarpa*.

This research aimed to investigate the difference between ER and PER, and whether the differential components of ER and PER could alleviate pain by inhibiting the contraction of mouse uterine smooth muscle cells. First, metabolomics methods based on UPLC-Q-TOF-MS were used to analyse the differential components of ER and PER. Afterwards, a dysmenorrhea mouse uterine smooth muscle cell model was established, and the effects of differential components on cell contraction and the expression of PGE2, ET-1, NO, P2X3, and Ca^2+^ in mouse uterine smooth muscle cells were explored (Figure 1). The research results demonstrated not only reveal the effect of the differential components on mouse uterine smooth muscle cells but also provide a theoretical basis for the quality control of ER and clinical application.

## 2. Materials and Methods

### 2.1. Reagents and Animals

Three batches of raw ER were used in this study. Raw ER was purchased from the First Hospital of Hunan University of Chinese Medicine (Hunan province, China). Prof. Yuming Zhang (The First Hospital of Hunan University of Chinese Medicine) identified the samples as *Euodia rutaecarpa (Juss.) Benth. Var. Bodinieri (node) Huang.*

Standard compounds such as 7-hydroxycoumarin and chlorogenic acid were provided by the National Institute for the Control of Pharmaceutical and Biological Products (Beijing, China). Limonin was provided by Chengdu Ruifensi Biotechnology Co. Ltd (Sichuan province, China). Estradiol benzoate injection was provided by Ningbo Sansheng Biotechnology Co., Ltd. (Zhejiang, Ningbo). The ELISA kits PGE2, ET-1, and NO were supplied by Shanghai Jingtian Biotechnology Co., Ltd. (Shanghai, China). P2X3 antibody and Furo-3/AM were provided by Beijing Bioss Biotechnology Co., Ltd. (Beijing, China). HCS CellMask Deep Red Stain was obtained from Thermo Fisher Scientific (Massachusetts, United States). The SAM antibody was provided by Affinity Biosciences (United States). Acetonitrile and methanol (MS-grade) were provided by Merck (Darmstadt, Germany). MS-grade formic acid was provided by Fisher (United States), and water was provided by Shenzhen Watsons Distilled Water Co., Ltd (Shenzhen, China).

10 ICR female mice in an SPF grade (weight of 18−20 g) were provided by Hunan Sadak Jingda Experiment Co., Ltd (Laboratory Animal Resource Center of Hunan Province, license number: SCXK [Xiang] 2016-0002). ICR mice were fed in the SPF laboratory with the First Hospital of Hunan University of Chinese Medicine for one week. All mice were free to obtain food and water. The laboratory is maintained at optimum temperature and humidity (20–26°C, 50%–70%).

### 2.2. Preparation of the Raw ER and PER Solutions

#### 2.2.1. PER Preparation

Yellow rice wine (30 mL) was diluted in 200 mL distilled water, and the diluted yellow rice wine was mixed with raw ER at a proportion of 1 : 1. The mixture was hermetically soaked for two hours until the wine was completely absorbed, after which it was stir-fried over a slow fire in a pan until the colour of raw ER deepened; next, it was removed and cooled to a temperature around 18–25°C.

#### 2.2.2. Raw ER and PER Extract Preparation

The three batches of raw ER and PER were weighed for 270 g, respectively. These samples were mixed with the water at a proportion of 1 : 10 and soaked in water for 30 min. After soaking, the mixture was decocted over a martial fire in a container until boiling, and then the fire turned into a gentle fire to be decocted for one hour. The liquid was filtered with gauze. The remaining residue was mixed with water at a proportion of 1 : 8; this method was repeated once. Twice the filtrates were mixed and condensed by a rotary evaporator. Finally, we obtained 75.57 g of raw ER and PER dry extract; namely, each gram of dry extract was equivalent to 3.62 g of TCM.

### 2.3. UPLC-Q-TOF-MS Analysis

#### 2.3.1. Chromatographic Conditions

The chromatographic column of the Agilent ZORBAX Eclipse Plus C18 (3.0 × 100 mm, 1.8 *μ*m) was used, and the flow rate was 0.4 mL/min. The mobile phase was composed of acetonitrile (A) and water containing 0.1% formic acid (B), with an injection volume of 1 *μ*L. A gradient elution procedure was used: 0–10 min, 5%–15% A; 10–15 min, 15%–20% A; 15–25 min, 25%–45% A; and 25–40 min, 45%–80% A.

#### 2.3.2. Mass Spectrometry Conditions

The mass spectrometer was equipped with an ESI. The scanning detection was approximately M/Z100–1700, the solvent gas was nitrogen at 6.8 L/min, the desolventizing temp was 325°C, and the source temp was 350°C. The voltage of the capillary was adjusted to 40 V.

#### 2.3.3. UPLC-Q-TOF-MS Data Processing

The collected metabonomics data were turned into mass/charge ratio (*m*/*z*) data using MassHunter Profinder software, which can perform peak extraction, recognition, matching, alignment, and normalization. The datasets, composed of sample name, retention time, mass charge ratio, and peak area, were derived from the software, and all *m*/*z* values were normalized. Afterwards, the SIMCA-P 14.1 statistical software was applied to multivariate statistical analysis (PCA and OPLS-DA), which was used to screen differential compounds. The screening conditions were as follows: (VIP) value > 1, P (CORR) > 0.05, fold change ≥ 1.4, and *t*-test *P* < 0.05. The information on the differential metabolites was input into Qualitative Analysis software from the Agilent Technologies Scientific company for recognition. Determination of the differential metabolites in ER and PER was done by using accurate mass spectrometry.

### 2.4. Inhibitory Effect of Differential Components on Dysmenorrhea Mouse Uterine Smooth Muscle Cells

#### 2.4.1. Dysmenorrhea Mice Model

10 ICR mice were numbered in sequence and randomly divided into two groups: normal group (*n* = 4) and dysmenorrhea mice model group (*n* = 6). The table of random numbers was used to randomize. Each group of mice was raised in a cage and named, and each mouse was regarded as an experimental unit. Apart from the director, the blinded method was useful for all researchers until the end of the experiment, and the experimental dates were performed independently by two participants. Since estradiol benzoate can thicken the endometrium and increase uterine smooth muscle contraction. The model group mice were subcutaneously injected with 10 mg/kg of estradiol benzoate for ten days, the normal group was saline. Meanwhile, all mice should be monitored for 30 minutes after each intervention. The animals were included in the later cell experiment if the animal was to survive, otherwise it was eliminated. All 10 ICR mice were entered into the later cell experiment. On the 10^th^ day, mice were euthanized by carbon dioxide asphyxiation. The animal experiment was performed via a protocol approved by the Experimental Animal Ethics Committee of the First Hospital of Hunan University of Chinese Medicine (NO: ZYFY20211030), in accordance with the Guide for the National Standards of the People's Republic of China (GB-T 1.1–2009).

#### 2.4.2. Cell Culture, Identification, and Grouping

The uteri were isolated from dysmenorrhea and normal mice and soaked in 75% alcohol for 30 s. The fat and connective tissue was removed from the separated uterine tissue surface. The mouse uterine strips were cut into 3–5 mm pieces with ophthalmic scissors and incubated in DMEM containing 0.02% collagenase II and 15% serum for 30 min at 37°C. The collagenase digestion solution was removed after centrifugation. The separated cells were resuspended in a DMED containing fetal bovine serum. On the second day, the separated cells were moved to the cell culture bottle for incubation at 37°C and 5% CO_2_. Half of the medium was then replaced with a culture medium every two days.

Primary cultures of dissociated cells were performed as we have described. While these cells were grown to 100% confluence, they will interact with trypsin in the cell culture bottle. Then, these cells and trypsin in the cell culture bottle were isolated by centrifugal machines. The separated cells were resuspended in a 96-well cell culture plate with a CO_2_ cell culture medium at a ratio of 10,000 per well for 48 h. PBS was then used to wash the cell culture plate twice. All cells were fixed with 4% cell fixative for 45 min, treated with 0.25% triton-100 for 15 min, and blocked with 5% BSA for 10 min. Afterwards, these cells were incubated in antibodies against the *α*-SMA diluent (1 : 300) at 4°C for overnight and the goat anti-rabbit Alexa Fluor ® 488 Conjugate (1 : 400) for 30 min at 37°C. PBS was used to wash the cell culture plate five times. These cells were then incubated in the dark for 12 min at room temperature with DAPI (1 : 800), and the morphology of dysmenorrhea mouse uterine smooth muscle cells was observed by a laser confocal microscope.

The isolated dysmenorrhea uterine smooth muscle cells were randomly divided into four groups: model group, 7-hydroxycoumarin group (1 mmol/L), chlorogenic acid (1 mmol/L), and limonin (50 *μ*mol/L). The normal group consisted of the isolated normal uterine smooth muscle cells, which were repeated 3 times in each group. We have performed a series of preliminary concentration experiment, which determined the optimum concentration of these compounds (see supplementary file (available here)).

#### 2.4.3. ELISA Assay

After administering for 24 h and collecting the supernatant, the cell supernatant was centrifuged at 3000 ×g for 15 min at room temperature. According to the instructions, the concentration of PGE2, ET-1, and NO in the uterine smooth muscle in each group was measured using the PGE2, ET-1, and NO ELISA kit.

#### 2.4.4. Immunofluorescence Staining

All cells were fixed with 4% cell fixative for 45 min, treated with 0.25% triton-100 for 15 min, and blocked with 5% BSA for 10 min. Afterwards, these cells were incubated in the cell culture plate containing 100 *μ*L HCS CellMask Deep Red Stain at room temperature in the dark for 30 min. The level of cell contraction was detected by a laser confocal microscope.

After being administered for 24 h, each group cells were washed twice with calcium-free D-hank's solution and then incubated in the cell culture plate containing 5 *μ*mol/L Furo-3/AM working solution at 37°C in the dark for 45 min. A laser confocal microscope was used for detection of the Ca^2+^ level.

We detected the expression of the P2X3 protein using a laser confocal microscope. All cells were fixed with 4% cell fixative for 45 min, treated with 0.25% triton-100 for 15 min, and blocked with 5% BSA for 10 min. Afterwards, these cells were incubated in primary antibody in P2X3 diluent (1 : 200) overnight at 4°C and then the goat anti-rabbit ALEXA Flour® 488 Conjugate (1 : 400) for 30 min at 37°C. After washing five times with PBS, all cells were incubated with DAPI (1 : 800) in the dark for 12 min at room temperature.

#### 2.4.5. Statistical Analysis

All experimental data were processed by Graphpad prism software. The calculation results were expressed as means ± standard deviations. The primary outcomes are the uterine smooth muscle cell contraction. Other indicators are secondary results. Student's *t*-test was used to compare the differences between the two groups. One-way ANOVA was used for the comparison of multiple groups and Tukey's test was the post hoc test. Shapiro–Wilk test was used for normality assessment test, and all groups satisfy the normal distribution (see supplementary file (available here)). There was a significant difference (*P* < 0.05).

## 3. Results

### 3.1. Metabolomics Data Analysis

#### 3.1.1. PCA Analysis of Raw ER and PER

**Figure 2:**
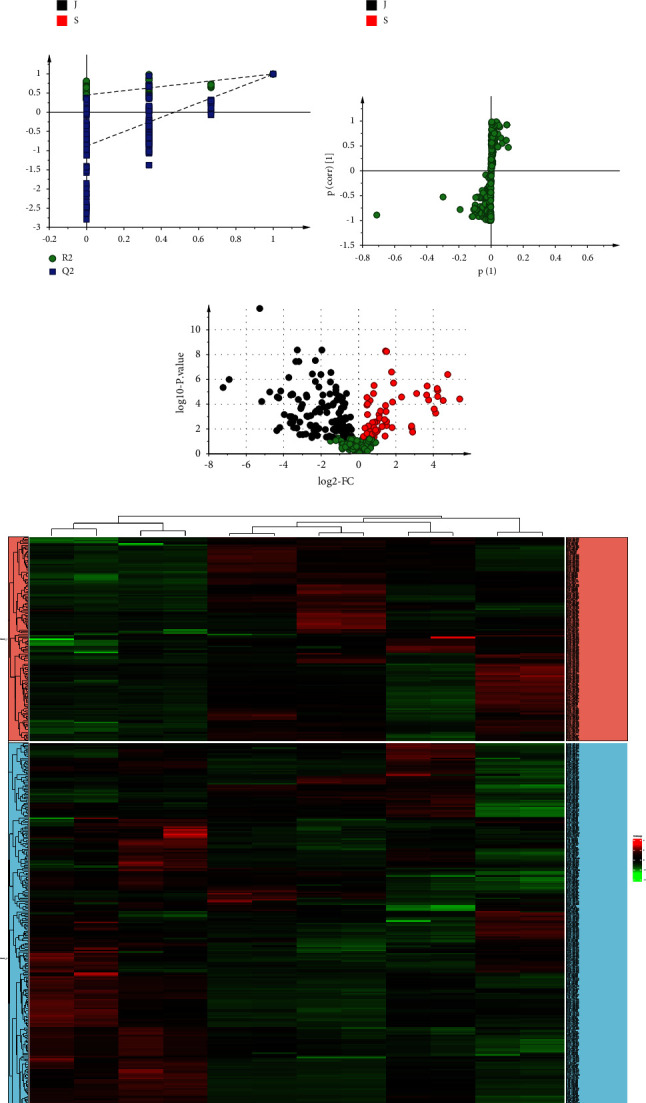
The metabonomics analysis of Raw ER and PER. (a) PCA score plot in positive ion mode. (b) OPLS score plot in positive ion mode. (c) Validate model of OPLS-DA. (d) S-plot analysis of Raw ER and PER. (e) Volcanic map of Raw ER and PER. (f) Heatmap analysis of Raw ER and PER. *Note*. WZY-S: raw ER; WZY-J: PER.

We used PCA to analyse the sample data for raw ER and PER. In the positive ion, the PCA model parameter *R*_2_*X* (representing the PCA model interpretation rate) was 0.946, indicating that the model was stable. The sample points of the raw ER and PER group were completely separated in the t [1] line (Figure 2(a)). The results suggested that the chemical components before and after ER wine processing had significant differences. As shown in t [2] line, the points, WZY-S-1-1, WZY-S-1-2, WZY-S-2-1, WZY-S-2-2, WZY-J-1-1, and WZY-J-1-2, are gathered in the positive semi-axis. The points of WZY-S-3-1, WZY-S-3-2, WZY-J-2-1, WZY-J-2-2, WZY-J-3-1, and WZY-J-3-2 are gathered in the negative half axis (Figure 2(a)), which indicates that the chemical constituents of ER were also different among batches of the same variety.

#### 3.1.2. OPLS-DA Analysis of Raw ER and PER

As illustrated in the OPLS-DA score plots (Figure 2(b)), raw ER and PER were completely separated in the positive ion. Meanwhile, we performed permutation tests 200 times and repeated cross-validation (2CV) one time to evaluate the reasonableness of the established OPLS-DA model (Figure 2(c)). In the positive ion, *R*_2_*Y* = 0.997 and *Q*_2_*X* = 0.99, indicating that 99.7% of the samples were in accordance with pattern recognition, and the model's predictive accuracy was 99% (Figures 2(b) and 2(d)). These consequences indicate that the model had preferable fitting precision and predictability.

#### 3.1.3. Volcanic Map

The volcanic map can also show the difference between raw ER and PER, where the abscissa is log2 (difference multiple) and the ordinate is −log10 (*Q*-value). The spot with a fold change ≤1.4 and a *Q*-value <0.05 was meant as black; the spot with a fold change ≥1.4 and a *Q*-value <0.05 was meant as red; the others are green (Figure 2(e)). The results may demonstrate that there are differences between raw ER and PER.

#### 3.1.4. Heatmap Analysis

The changes in raw ER and PER were shown visually by Heatmap analysis (Figure 2(f)). The abscissa axis was different batches of raw ER and PER group; the ordinate axis was the compounds of the corresponding groups. The results showed that the compounds of the raw ER group were different from those of the PER group and different among batches of the same variety, further considering that the treatment actions of the raw ER group and PER were also different.

### 3.2. Differential Composition Analysis of Raw ER and PER

**Figure 3:**
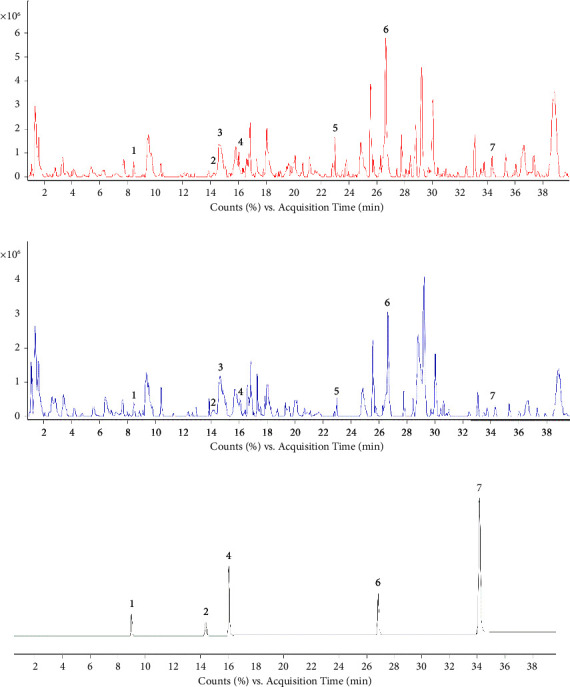
Total ion chromatograms (TIC) of raw ER and PER in positive ions using UPLC-Q-TOF-MS. (a) The ion chromatograms of PER. (b) The ion chromatograms of raw ER. (c) The ion chromatograms of reference substances. *Note*. 1: chlorogenic acid; 2: 7-hydroxycoumarin; 3: hydroxy evodiamine; 4: laudanosine; 5: evollionines A; 6: limonin; 7: 1-methyl-2-[(z)-4-nonenyl]-4 (1H)-quinolone.

The ion chromatograms of raw ER, PER, and reference substances in the positive ion mode are shown in (Figures 3(a)–3(c)). According to the screening conditions VIP ≥ 1, |P (corr)| > 0.5, fold change ≥ 1.4, and *P* < 0.05, we found that the 7 differential compounds detected in the raw ER and PER, as follows: chlorogenic acid, 7-hydroxycoumarin, hydroxy evodiamine, laudanosine, evollionines A, limonin, 1-methyl-2-[(z)-4-nonenyl]-4 (1H)-Quinolone (Table 1). Among then, the reference substances of chlorogenic acid, 7-hydroxycoumarin, laudanosine, limonin, 1-methyl-2-[(z)-4-nonenyl]-4 (1H)-Quinolone are shown in Figure 3(c).

**Figure 4:**
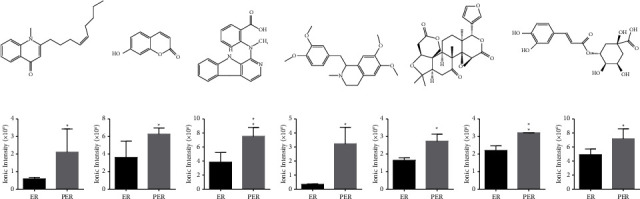
The chemical structure formulas and ionic intensity of differential compounds in the raw ER and PER. (a) The chemical structure formulas of six differential compounds. (b) The comparison of the ionic intensity of differential compounds.

As shown in Figure 4(a), the chemical structure formulas of 6 differential compositions were obtained from PubChem. In addition, the comparison of the ionic intensity of the markers in the raw ER and PER is shown in (Figure 4(b)). We found that the differential composition of ionic intensity were stronger in PER compared to raw ER.

### 3.3. Inhibitory Effects of 7-Hydroxycoumarin, Chlorogenic Acid, and Limonin on Dysmenorrhea Mouse Uterine Smooth Muscle Cells

#### 3.3.1. Morphology of Dysmenorrhea Mouse Uterine Smooth Muscle Cells

**Figure 5:**
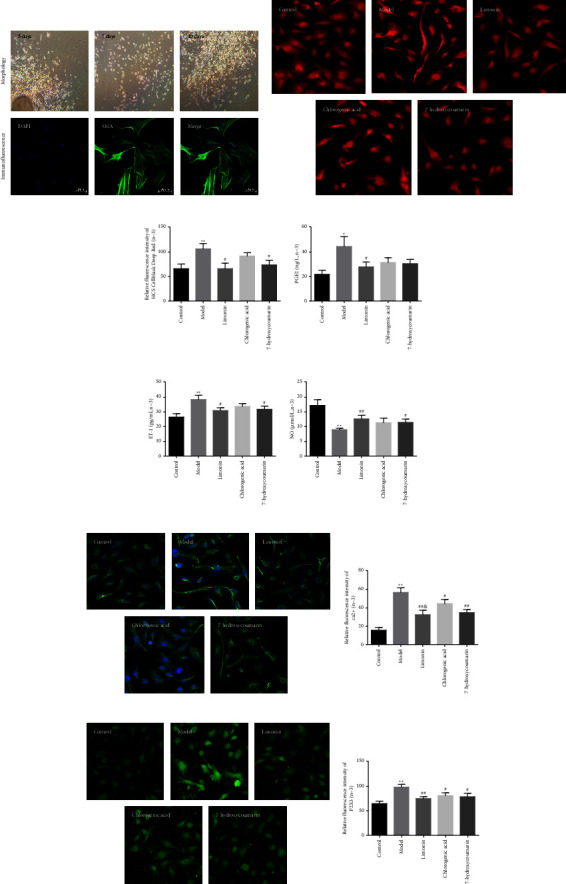
7-Hydroxycoumarin, chlorogenic acid, and limonin inhibit cell contraction, regulate the levels of NO, ET-1, and PGE2 and the expression of P2X3 and Ca^2+^ in dysmenorrhea mouse uterine smooth muscle cells. (a) Identification of dysmenorrhea mouse uterine smooth muscle cells (100 x). (b) Immunofluorescence staining of contraction in dysmenorrhea mouse uterine smooth muscle cells (200 x). (c) The level of cell contraction in dysmenorrhea mouse uterine smooth muscle cells. (d) The level of PGE2 in dysmenorrhea mouse uterine smooth muscle cells. (e) The level of ET-1 in dysmenorrhea mouse uterine smooth muscle cells. (f) The level of NO in dysmenorrhea mouse uterine smooth muscle cells. Figure (g) and (h) show the immunofluorescence and fluorescence intensity of Ca2+, respectively. Figure (i) and (j) show the immunofluorescence and fluorescence intensity of P2X3, respectively. *n* = 3; ^*∗∗*^*P* < 0.01 vs. normal. ^##^*P* < 0.01 vs. model group.

We used a laser confocal microscope to observe the morphology of dysmenorrhea mouse uterine smooth muscle cells. The results of cell imaging showed that the dysmenorrhea mouse uterine smooth muscle cells presented a spindle or irregular triangle with sharp processes. The cell nucleus was a circle or ellipse. These cells were arranged in parallel and partially overlapped in their growth phase. They showed a typical structure: “peak valley” (Figure 5(a)).

For further observation, immunofluorescence staining was used for dysmenorrhea mouse uterine smooth muscle cells. The immune reaction product was presented in green, and the expression of *α*-SMA was positive. More than 98% of the cells were mouse uterine smooth muscle cells in randomly selected visual fields. The morphological and immunofluorescence staining results showed that the cultured cells were mouse uterine smooth muscle cell (Figure 5(a)).

#### 3.3.2. 7-Hydroxycoumarin, Chlorogenic Acid, and Limonin Inhibited the Contraction of Dysmenorrhea Uterine Smooth Muscle Cells

To discuss the effect of 7-hydroxycoumarin, chlorogenic acid, and limonin on contraction in dysmenorrhea mouse uterine smooth muscle cell, we observed the dysmenorrhea mouse uterine smooth muscle cells contraction with Furo-3/AM fluorescent probe staining and laser confocal microscopy. The cell contraction was increased in the model group compared to the normal group, and the cell area was reduced (*P*=0.008, Figures 5(b) and 5(c) and Table 2). 7-Hydroxycoumarin could suppress the upregulation of cell contraction when compared to the model group (*P*=0.018, Figures 5(b) and 5(c) and Table 2). The application of limonin could inhibit cell contraction (*P*=0.011, Figures 5(b) and 5(c) and Table 2). The chlorogenic acid group could reduce the cell contraction, although not significantly. Notably, the cell contraction was not also found to have a significant difference between 7-hydroxycoumarin, chlorogenic acid, and limonin groups. The results indicated that 7-hydroxycoumarin, limonin, and chlorogenic acid could expand cells and inhibit the contraction of mouse uterine smooth muscle cells.

#### 3.3.3. 7-Hydroxycoumarin, Chlorogenic Acid, and Limonin Regulated the Levels of PGE2, ET-1, and NO in Dysmenorrhea Uterine Smooth Muscle Cells

Changes in PGE2, ET-1, and NO were associated with the pathogenesis of dysmenorrhea during menstruation. To investigate the effect of 7-hydroxycoumarin, chlorogenic acid, and limonin on PGE2, ET-1, and NO in dysmenorrhea mouse uterine smooth muscle cells. We measured the concentrations of these indices. The concentrations of PGE2 and ET-1 were increased in the model group compared to the normal group (*P*=0.011, *P*=0.006, respectively) (Figures 5(d) and 5(e), Tables 3, and 4), and NO was reduced (*P*=0.002, Figure 5(f) and Table 5). However, the concentration of PGE2 and ET-1 in the limonin group were suppressed compared to the model group (*P*=0.033, *P*=0.022, respectively) (Figures 5(d) and 5(e), Tables 3 and 4), and NO increased (*P*=0.009, Figure 5(f) and Table 5). 7-hydroxycoumarin had an influence on ET-1 and NO (*P*=0.036, *P*=0.025, respectively) (Figures 5(e) and 5(f), Tables 4 and 5). The chlorogenic acid group was not significant. There was no significant difference between the 7-hydroxycoumarin, chlorogenic acid, and limonin group. The results demonstrate that dysmenorrhea led to changes in PGE2, ET-1, and NO levels, and 7-hydroxycoumarin and limonin effectively regulated the levels of NO, ET-1, and PGE2 in dysmenorrhea mouse uterine smooth muscle cells.

#### 3.3.4. 7-Hydroxycoumarin, Chlorogenic Acid, and Limonin Inhibited the Expression of P2X3 and Ca^2+^ in Dysmenorrhea Uterine Smooth Muscle Cells

The P2X3-Ca^2+^ pathway is known to play a crucial role in pathological pain. For a further investigation of the effect of 7-hydroxycoumarin, chlorogenic acid, and limonin on dysmenorrhea, we measured the expression of P2X3 and Ca^2+^ with laser confocal microscopy. As shown in Figure 5 and Tables 6 and 7, the expression of P2X3 and Ca^2+^ was closed to normal levels at the treatment of 7-hydroxycoumarin (*P*=0.020, *P*=0.003, respectively) (Figures 5(g)–5(j)). The levels of P2X3-Ca^2+^ (*P*=0.004, *P*=0.004, respectively) (Figures 5(g)–5(j)) were also influenced by limonin, and chlorogenic acid could suppress the expression of P2X3 and Ca^2+^ (*P*=0.020, *P*=0.035, respectively) (Figures 5(g)–5(j)). Interestingly, the expression of Ca^2+^ shows a significant difference between the chlorogenic acid and limonin group (*P*=0.034, Figure 5(h)). These results suggest that 7-hydroxycoumarin, chlorogenic acid, and limonin effectively suppressed the expression of P2X3 and Ca^2+^ in dysmenorrhea mouse uterine smooth muscle cells. Meanwhile, the results of molecular docking show that the analgesic effects of 7-hydroxycoumarin, chlorogenic acid, and limonin may be related to the P2X3 receptor (see supplementary file (available here)).

## 4. Discussion

*Evodia rutaecarpa* (ER) was first recorded in *Shen Nong*'*s Herbal Classic* and has the features of warming and alleviating pain [19]. Increasing evidence has revealed that ER can alleviate dysmenorrhea by slowing the contraction frequency and intensity of uterine smooth muscle, and wine processing would strengthen the effect of dispelling cold to relieve pain [11, 20, 21]. However, few investigations have been conducted on the correlation between the analgesic effect and the active components of ER before and after wine processing. Therefore, this study used UPLC-Q-TOF-MS and metabolomics technology to screen and identify the differential components before and after ER wine processing, after which a dysmenorrhea mouse uterine smooth muscle cell model was established. The model was intervened with the differential components to examine its therapeutic effect and assess the underlying mechanism. Our research not only demonstrated wine processing can cause changes in components of raw ER but also found the differential components clearly attenuated dysmenorrhea mouse uterine smooth muscle cell contraction, reduced the levels of PGE2, ET-1, increased the content of NO, and suppressed the expression of P2X3 and Ca^2+^.

We finally identify seven differential components in the extracts of raw ER and PER: 7-hydroxycoumarin, chlorogenic acid, hydroxy evodiamine, laudanosine, limonin, evollionines A, and 1-methyl-2-[(z)-4-nonenyl]-4 (1H)-quinolone. Alkaloids are the main analgesic components of raw ER [22]. During processing, the infiltration of millet wine increases the permeability of decoction pieces and internal tissues, which is conducive to the dissolution of alkaloids and improves their dissolution efficiency [23]. Therefore, we speculated that the change in hydroxy evodiamine, laudanosine, and 1-methyl-2-[(z)-4-nonenyl]-4 (1H)-quinolone is related to wine processing. The analgesic effects of 7-hydroxycoumarin, chlorogenic acid, and limonin have been reported in relevant studies. Barros TA found that 7-hydroxycoumarin can produce sustained analgesic effects on chronic inflammatory pain induced by acetic acid and formalin [24]. Bagdas suggested that chlorogenic acid has antinociceptive effects on streptozotocin-induced diabetic neuropathic pain in rats [25]. The limonin derivative V-A-8's analgesic and anti-inflammatory effects were more powerful than aspirin and naproxen [26]. Moreover, we found that the analgesic effect of Evodia rutaecarpa is related to 7-hydroxycoumarin, chlorogenic acid, and limonin by molecular docking technology. Therefore, 7-hydroxycoumarin, chlorogenic acid, and limonin were selected for validation in the later experimental validation.

ER has a good curative effect on dysmenorrhea [27]. However, there have been few reports on the effect and mechanism of its differential components before and after wine processing on dysmenorrhea. Therefore, we have cultured dysmenorrhea mouse uterine smooth muscle cells and intend to use this model to evaluate the effect of differential components before and after ER wine was processed (7-hydroxycoumarin, chlorogenic acid, and limonin) on dysmenorrhea mouse uterine smooth muscle cell contraction. Consistent with other reports, our study demonstrated that 7-hydroxycoumarin, chlorogenic acid, and limonin could inhibit the contraction of mouse uterine smooth muscle cells, which suggested that the differential components may be effective in inhibiting uterine smooth muscle contraction.

In addition, the endocrine factors PGE2, ET-1, and NO play crucial roles in dysmenorrhea [28–30]. During menstruation, the increase in PGE2 content destroys endometrial cells and releases a large amount of prostaglandinE2*α,* which may cause dysmenorrhea as a result of strong contraction of uterine smooth muscle [31]. ET-1 and NO are two vasoactive substances with opposing effects on the endometrium [32]. The increase in ET-1 content causes blood vessels to contract strongly and produce pain [32]. In dysmenorrhea, different doses of NO act on target cells and exert two-way adjusting effects. When the NO content is increased, it can inhibit the release of nociceptive substances and alleviate pain; by contrast, it will promote the contraction of uterine smooth muscle cells and cause pain [32]. As described in Figure 5, the levels of PGE2 and ET-1 were suppressed in the 7-hydroxycoumarin and limonin groups, and the content of NO was upregulated. In addition, the level of PGE2, ET-1, and NO in the chlorogenic acid group changed compared to the model group, but there was no significant difference, which suggested that 7-hydroxycoumarin, limonin, and chlorogenic acid regulated the level of endocrine factors in uterine smooth muscle cells and thus improved dysmenorrhea.

The P2X3 receptor plays an essential role in various pain symptoms [33]. Activation of the P2X3 receptor can cause upregulation of Ca^2+^ levels in rats with chronic pancreatitis, and the antagonist treatment downregulates P2X3 activity and Ca^2+^ signal transduction [34]. The pathophysiology of dysmenorrhea may involve abnormalities within the critical effector systems responsible for Ca^2+^ effects on uterine smooth muscle cells [35]. Reports have demonstrated that calcium in smooth muscle cells regulates cell contraction as a second messenger, and the cell contraction may be associated with an increase in intracellular calcium concentration [36, 37]. In addition, the P2X3 receptor antagonist A-317491 has a positive effect in the treatment of hyperalgesia caused by endometriosis in rats. Furthermore, researches have reported that the P2X3-Ca^2+^ pathway is involved in ATP-induced analgesia actions [38]. Our study demonstrated that 7-hydroxycoumarin, chlorogenic acid, and limonin could inhibit dysmenorrhea by inducing downregulation of P2X3 and Ca^2+^.

The limitations of our experiment are as follows: (1) we selected a small animal sample size because the original intention of the study was to obtain uterine smooth muscle cells by establishing a dysmenorrhea mouse model. (2) “A priori” power calculations in the study were lacking, which may have made the sample size insufficient and influenced the interpretation of the experimental data.

## 5. Conclusion

In conclusion, seven compounds were screened and identified in the extracts of raw ER and PER, and 7-hydroxycoumarin, chlorogenic acid, and limonin were able to inhibit cell contraction and the expression of PGE2, ET-1, P2X3, and Ca2^+^ in dysmenorrhea mouse uterine smooth muscle cells. Furthermore, these compounds increased NO content. These results demonstrated the compounds of the PER were different from those of the raw ER, and 7-hydroxycoumarin, chlorogenic acid, and limonin could improve dysmenorrhea mice's uterine smooth muscle cell contraction and relieve pain when combined with endocrine factors and the P2X3-Ca2^+^ pathway.

## Figures and Tables

**Table 1:** Differential components identification of ER samples.

Number	T (min)	Name	Molecular formula	Molecular weight [M-H]+	Error	Fragment ion
1	8.02	Chlorogenic acid	C16H18O9	355.0984	8.93	163, 145
2	14.35	7-Hydroxycoumarin	C9H6O3	163.0858	3.37	135, 121
3	14.98	Hydroxy evodiamine	C24H23N3O8	482.2598	−0.91	303, 174
4	16.06	Laudanosine	C21H27NO4	358.2016	−0.02	174.091
5	23.52	Evollionines A	C19H15N3O2	318.1232	1.11	257, 285, 300
6	26.77	Limonin	C26H30O8	471.7579	−0.3	425, 339, 161
7	34.16	1-Methyl-2-[(z)-4-nonenyl]-4 (1H)-Quinolone	C19H25NO	284.2013	−1.39	221, 173, 121

**Table 2:** Details of the descriptive statistics and 95% confidence intervals for cell contraction.

Group	*N*	Mean	Standard deviation	Lower 95% confidence intervals	Upper 95% confidence intervals	Minimum	First quartile	Median	Third quartile	Maximum
Normal group	3	66.17	9.46	42.67	89.67	58.61	58.61	63.12	76.78	76.78
Model group	3	106.7	10.95	79.37	133.8	94.41	94.41	109.70	115.70	115.70
Limonin group	3	65.94	11.48	37.43	94.45	53.19	53.19	69.18	75.45	75.45
Chlorogenic acidgroup	3	91.98	6.68	75.38	108.60	86.71	86.71	89.73	99.49	99.49
7-Hydroxycoumarin group	3	73.83	9.67	49.81	97.84	63.02	63.02	76.81	81.65	81.65

**Table 3:** Details of the descriptive statistics and 95% confidence intervals for PGE2.

Group	*N*	Mean	Standard deviation	Lower 95% confidence intervals	Upper 95% confidence intervals	Minimum	First quartile	Median	Third quartile	Maximum
Normal group	3	21.74	3.40	13.31	30.16	18.50	18.50	21.44	25.26	25.26
Model group	3	44.48	8.06	24.47	64.49	37.32	37.32	42.91	53.21	53.21
Limonin group	3	27.91	3.96	18.08	37.74	24.68	24.68	26.74	32.32	32.32
Chlorogenic acidgroup	3	31.44	3.98	21.56	41.32	27.62	27.62	31.15	35.56	35.56
7-Hydroxycoumarin group	3	30.46	3.60	21.53	39.39	27.32	27.32	29.68	34.38	34.38

**Table 4:** Details of the descriptive statistics and 95% confidence intervals for ET-1.

Group	*N*	Mean	Standard deviation	Lower 95% confidence intervals	Upper 95% confidence intervals	Minimum	First quartile	Median	Third quartile	Maximum
Normal group	3	26.51	2.42	20.51	32.51	24.89	24.89	25.35	29.29	29.29
Model group	3	38.38	2.96	31.03	45.73	35.35	35.35	38.53	41.26	41.26
Limonin group	3	30.95	1.97	26.06	35.85	28.68	28.68	32.02	32.17	32.17
Chlorogenic acidgroup	3	33.83	1.84	29.25	38.41	31.71	31.71	34.74	35.05	35.05
7-Hydroxycoumarin group	3	31.86	2.12	26.59	37.1	29.44	29.44	32.77	33.38	33.38

**Table 5:** Details of the descriptive statistics and 95% confidence intervals for NO.

NO	*N*	Mean	Standard deviation	Lower 95% confidence intervals	Upper 95% confidence intervals	Minimum	First quartile	Median	Third quartile	Maximum
Normal group	3	17.23	1.97	12.34	22.13	14.99	14.99	18.02	18.68	18.68
Model group	3	8.95	0.49	7.73	10.16	8.51	8.50	8.87	9.47	9.47
Limonin group	3	12.64	1.24	9.56	15.73	11.59	11.59	12.32	14.02	14.02
Chlorogenic acidgroup	3	11.33	1.60	7.37	15.29	9.90	9.90	11.05	13.05	13.05
7-Hydroxycoumarin group	3	11.45	1.13	8.64	14.27	10.62	10.62	10.99	12.75	12.75

**Table 6:** Details of the descriptive statistics and 95% confidence intervals for P2X3.

Group	*N*	Mean	Standard deviation	Lower 95% confidence intervals	Upper 95% confidence intervals	Minimum	First quartile	Median	Third quartile	Maximum
Normal group	3	64.54	5.14	51.76	77.32	59.62	59.62	64.12	69.88	69.88
Model group	3	98.39	5.75	84.10	112.70	92.32	92.32	99.08	103.80	103.80
Limonin group	3	75.10	3.85	65.53	84.67	72.60	72.60	73.17	79.54	79.54
Chlorogenic acidgroup	3	81.23	5.47	67.64	94.81	76.45	76.45	80.04	87.19	87.19
7-Hydroxycoumarin group	3	78.62	7.12	60.92	96.32	70.46	70.46	81.80	83.60	83.60

**Table 7:** Details of the descriptive statistics and 95% confidence intervals for Ca^2+^.

Group	*N*	Mean	Standard deviation	Lower 95% confidence intervals	Upper 95% confidence intervals	Minimum	First quartile	Median	Third quartile	Maximum
Normal group	3	15.60	3.15	7.79	23.41	12.46	12.46	15.59	18.75	18.75
Model group	3	56.85	5.05	4.31	69.39	51.53	51.53	57.94	61.27	61.27
Limonin group	3	32.54	4.83	20.55	44.53	28.65	28.65	31.02	37.94	37.94
Chlorogenic acidgroup	3	44.73	4.41	33.87	55.69	41.39	41.39	43.08	49.73	49.73
7-Hydroxycoumarin group	3	34.92	3.25	26.84	42.99	31.40	31.40	35.54	37.81	37.81

## Data Availability

The data used in this study are made available upon reasonable request to the corresponding author.

## Supplementary Materials

Supplementary Materials The maximum nontoxic concentration, molecular docking between P2X3, 7-hydroxycoumarin, chlorogenic acid, and limonin, the preliminary concentration experiment, and the normality assessment test were included in the supplementary file.
