# Supplementary material for: Mechanism and Pharmacodynamic Substance Basis of Raw and Wine-Processed Evodia rutaecarpa on Smooth Muscle Cells of Dysmenorrhea Mice
Source: Pain Res Manag. 2023 Jun 1;2023:7711988. doi: 10.1155/2023/7711988 (PMC10250099; doi:10.1155/2023/7711988)
Supplement: Supplementary Materials — The maximum nontoxic concentration, molecular docking between P2X3, 7-hydroxycoumarin, chlorogenic acid, and limonin, the preliminary concentration experiment, and the normality assessment test were included in the supplementary file. [file 7711988.f1.pdf]

1.Biological activity assay

To elucidate the efficacy of the differential compounds against dysmenorrhea mice uterine smooth muscle cell contraction, we first assessed the cytotoxicity of the differential compounds on the growth of dysmenorrhea mice uterine smooth muscle cells. The results showed that the differential compounds had no toxicity to dysmenorrhea mice uterine smooth muscle cells between 1000μM and 10μM (Figure 1, Table 1).

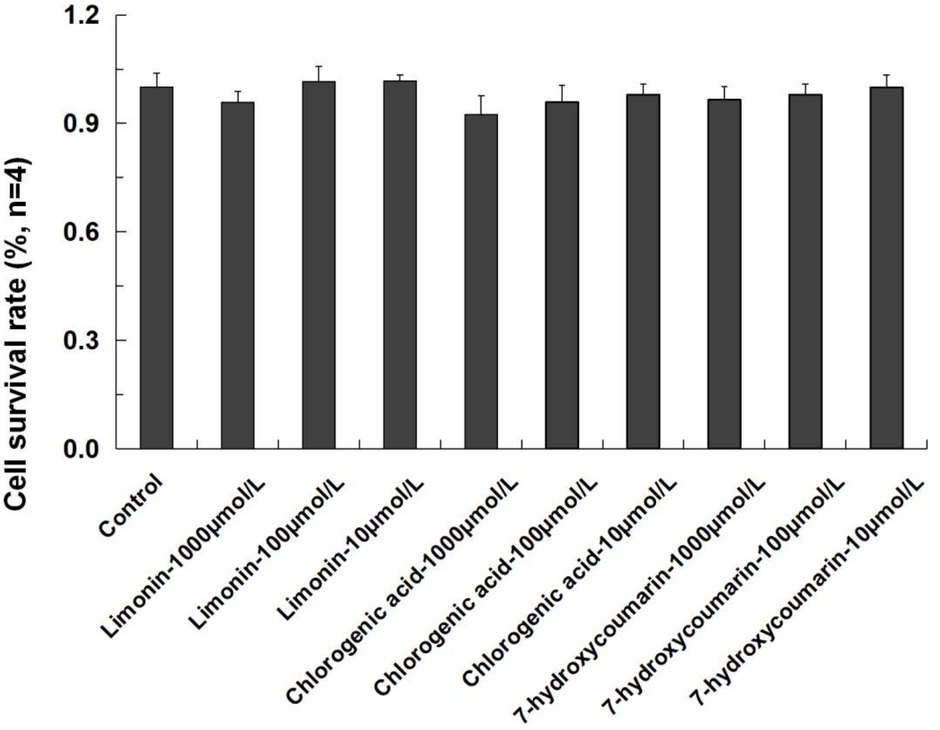

FIGURE 1:Compounds had no cellular toxicity on dysmenorrhea mice uterine smooth muscle cells between 1000μM and 10μM. Data are presented as mean±SD.

TABLE 1: Cell viability of dysmenorrhea mice uterine smooth muscle cells with different concentrations of compounds.

Compounds	Drug concentration(μM)	Mean±SD
Control	-	100±3.85
	1000	98.02±3.09
Limonin	100	101.53±4.31
	10	101.77±1.09
	1000	95.42±5.27
Chlorogenic acid	100	95.85±4.62
	10	97.93±2.89
	1000	96.50±3.74
7-hydroxycoumarin	100	97.89±2.95
	10	99.93±3.50

2.Verification of molecular docking between active ingredients and core targets

P2X3 receptor is involved in pain transduction in peripheral nervous system and

central nervous system. To verify whether the correlation between the differential compounds and the regulation of P2X3 via molecular docking.The binding energy ≤ −5.0 kJ/mol was selected as the screening condition. The results showed that the differential component has great bonding with P2X3 and may be effects its function through hydrogen bonding (Figure 2, Table 2).

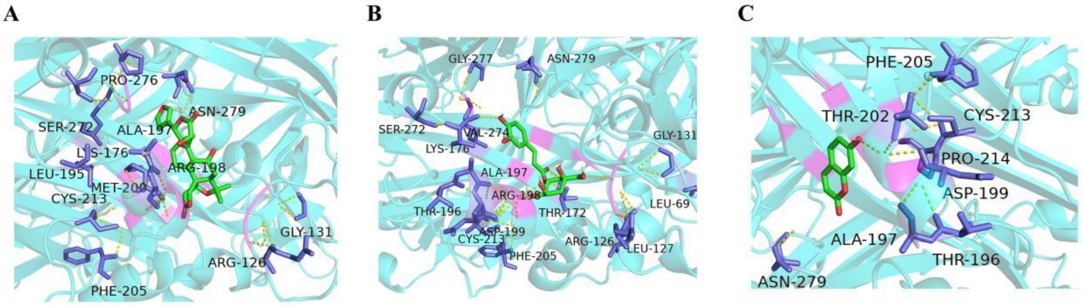

PDB ID	Core target	Binding Energy/ (kcal/mol)		
		Limonin	Chlorogenic acid	7-hydroxycoumarin
6AH5	P2X3	-7.7	-6.8	-5.8

FIGURE 2: P2X3 docking diagram with key active ingredients

TABLE 2: Minimum binding energy of the active component to the core target molecule

3. The normality assessment test

Shapiro-Wilk test was used for normality assessment test, and all groups satisfy the normal distributio (Figure 3-8)

正态性检验

	组别	柯尔莫戈洛夫-斯米诺夫[a] 统计	自由度	显著性	夏皮洛-威尔克 统计	自由度	显著性
强度	正常组	.211	3	.	.991	3	.815
	模型组	.313	3	.	.894	3	.367
	7-羟基豆素	.307	3	.	.903	3	.395
	绿原酸	.213	3	.	.990	3	.807
	柠檬苦素	.313	3	.	.895	3	.368

a. 里利氏显著性修正

Figure 3 The normal distribution of cell contraction

正态性检验

	组别	柯尔莫戈洛夫-斯米诺夫[a] 统计	自由度	显著性	夏皮洛-威尔克 统计	自由度	显著性
强度	正常组	.201	3	.	.994	3	.856
	模型组	.244	3	.	.972	3	.676
	7-羟基豆素	.253	3	.	.964	3	.637
	绿原酸	.196	3	.	.996	3	.878
	柠檬苦素	.284	3	.	.934	3	.503

a. 里利氏显著性修正

Figure 4 The normal distribution of PGE2

正态性检验

	组别	柯尔莫戈洛夫-斯米诺夫[a]			夏皮洛-威尔克		
		统计	自由度	显著性	统计	自由度	显著性
强度	正常组	.351	3	.	.827	3	.180
	模型组	.187	3	.	.998	3	.915
	7-羟基豆素	.333	3	.	.862	3	.274
	绿原酸	.356	3	.	.818	3	.157
	柠檬苦素	.372	3	.	.783	3	.073

a. 里利氏显著性修正

Figure 5 The normal distribution of ET-1

正态性检验

	组别	柯尔莫戈洛夫-斯米诺夫[a]			夏皮洛-威尔克		
		统计	自由度	显著性	统计	自由度	显著性
强度	正常组	.322	3	.	.880	3	.325
	模型组	.232	3	.	.980	3	.726
	7-羟基豆素	.326	3	.	.874	3	.307
	绿原酸	.237	3	.	.976	3	.705
	柠檬苦素	.269	3	.	.949	3	.567

a. 里利氏显著性修正

Figure 6 The normal distribution of NO

正态性检验

	组别	柯尔莫戈洛夫-斯米诺夫[a]			夏皮洛-威尔克		
		统计	自由度	显著性	统计	自由度	显著性
强度	正常组	.199	3	.	.995	3	.865
	模型组	.215	3	.	.989	3	.800
	7-羟基豆素	.359	3	.	.811	3	.141
	绿原酸	.253	3	.	.965	3	.639
	柠檬苦素	.339	3	.	.851	3	.242

a. 里利氏显著性修正

Figure 7 The normal distribution of P2X3

正态性检验

	组别	柯尔莫戈洛夫-斯米诺夫[a]			夏皮洛-威尔克		
		统计	自由度	显著性	统计	自由度	显著性
强度	正常组	.175	3	.	1.000	3	.995
	模型组	.252	3	.	.965	3	.642
	7-羟基豆素	.243	3	.	.972	3	.681
	绿原酸	.313	3	.	.895	3	.368
	柠檬苦素	.290	3	.	.926	3	.474

a. 里利氏显著性修正

Figure 8 The normal distribution of Ca^{2+}

正态性检验

		柯尔莫戈洛夫-斯米诺夫[a]			夏皮洛-威尔克		
	组别	统计	自由度	显著性	统计	自由度	显著性
强度	正常组	.175	3	.	1.000	3	.995
	模型组	.252	3	.	.965	3	.642
	7-羟基豆素	.243	3	.	.972	3	.681
	绿原酸	.313	3	.	.895	3	.368
	柠檬苦素	.290	3	.	.926	3	.474

a. 里利氏显著性修正

4. The preliminary concentration experiment

we have studied the pilot study of drug concentration. Based on these references, A drug concentration gradient was used, as follow: 1000μmol/L, 100μmol/L, 10μmol/L, 1μmol/L. Each cell group was repeated three times. The result showed that the effect of 7-hydroxycoumarin and chlorogenic acid were best to uterine smooth muscle cell contraction in 1000μmol/L, limonin was between 100μmol/Land 10μmol/L. The concentration of limonin was determined to be 50μmol/L according to the references (Table 3).

Table 3: Cell contraction of dysmenorrhea mice uterine smooth muscle cells with different concentrations of compounds.

Group	Drug concentration(μmol/L)	Mean±SD
Normal group	-	67.17±5.86
Model group	-	108.24±2.50
7-Hydroxycoumarin group	1000	67.92±2.11
	100	80.37±4.56
	10	90.80±5.57
	1	95.62±5.27
chlorogenic acid group	1000	91.64±3.33
	100	95.16±4.46
	10	97.92±5.44
	1	103.78±3.49
Limonin group	1000	81.07±3.88
	100	69.61±4.17
	10	69.03±3.26
	1	96.20±4.90
